# Performance-Based Accountability and Teacher Emotions: Role of *Zhongyong* Thinking

**DOI:** 10.3389/fpsyg.2021.612206

**Published:** 2021-04-13

**Authors:** Ying Zhang, Kwok Kuen Tsang

**Affiliations:** College of Educational Administration, Faulty of Education, Beijing Normal University, Beijing, China

**Keywords:** performance-based accountability, teacher emotions, *zhongyong*, thinking style, China

## Abstract

Teachers’ emotions may be affected by structural reforms of education that emphasizes performance-based accountability (PBA) and by individual psychological processes like thinking style, but there is a lack of research concerning the relationship between the three factors. In this study, thus, we attempted to test the influence of PBA on teacher emotions and to examine whether the relationship was moderated by a *zhongyong* thinking (ZYT) style in a Chinese context. A sample of 402 primary and secondary schoolteachers from Hubei, Liaoning, and Beijing in China participated in this study. Structural equation modeling was applied to develop moderation models. The results demonstrated that PBA is a singificant factor with respect to teachers’ joy, sadness/frustration, anger, and fear, as related to their job of teaching, but not love of their profession. Moreover, the ZYT style may moderate the relationship between PBA and joy.

## Introduction

Teaching has been regarded as emotional labor, implying that teachers need to be emotionally committed to nurturing students (Yin et al., 2019). If teachers are charged with positive emotions, their work effectiveness and psychological well-being are enhanced and vice versa (Day and Qing, 2009). Nevertheless, previous studies illustrate that around the world, teacher emotions tend to be drained by managerial education reforms because reforms introduce market logic, such as performance-based accountability (PBA), into public schooling and profoundly impact teachers’ work life (Tsang, 2019). In Australia, for instance, PBA has contributed to increased stress, pressure, and anxiety for teachers (Thompson, 2013). A review of Ro (2019) also reveals that teachers in America and Singapore experience stress and frustration caused by accountability mandates. As more attention has been given to negative teacher emotions engendered by PBA worldwide, little empirical research has focused on Chinese teachers’ emotional response to PBA.

Since market-oriented education reforms were initiated in the 1980s in China, a series of policies aimed at holding teachers accountable for student performance have profoundly influenced teachers (Wong, 2008; Lo et al., 2015). For example, the performance-linked teacher salary reform implemented in 2009 emphasizes the principle of “good performance, good pay” and has degraded teachers to performative workers with an approach of “compliant professionalism” (Wang et al., 2014). As a result, Chinese teachers tend to feel stress and anxiety in this PBA context. Despite the negative effects of PBA, there is also a long-term cultural recognition of test scores among Chinese individuals because China has been an examination-oriented society since imperial China set up examination systems to select government officials a 1000 years ago (Miyazaki, 1974). In an examination-oriented society, people generally consider the test score to be an important indicator to judge students’ abilities and attainment that further reflects teacher quality. In this regard, how PBA influences teacher emotions in China could be more complex and even paradoxical to other societies in the world.

However, the literature suggests that teachers’ emotions are affected not only by structural reforms but also by individual psychological processes, such as thinking style (Tsang, 2019). As influenced by Confucianism, the *zhongyong* thinking (ZYT) style has become a far-reaching thinking style affecting the Chinese population (Ames and Hall, 2001). In general, ZYT tends to make the Chinese “consider things carefully from different perspectives, avoid going to extremes, behave in situationally appropriate ways, and maintain harmony” (Ji et al., 2010). In this regard, Chinese teachers with high ZYT are more likely to interpret both good and bad PBA holistically and avoid expressing extreme emotions either positively or negatively. Accordingly, ZYT may affect the relationship between PBA and teacher emotions, but there is a lack of research concerning the relationship between the three factors.

Therefore, the present study attempts to fill the gaps in the literature by examining the relationship between teacher emotions, PBA, and ZYT in China. First, although many studies suggest negative teacher emotions engendered by PBA worldwide, there is little knowledge about Chinese teachers’ emotions toward PBA in a special examination-oriented cultural context. Therefore, the research findings may advance knowledge of the influence of PBA on teacher emotions in a global context. Second, there is a lack of studies taking individual psychological processes into account when examining the relationship between teacher emotions and structural reforms. Thus, considering both PBA and ZYT simultaneously would be helpful to gain a comprehensive understanding of emotional arousal theoretically. Moreover, similar to Chinese societies, many East Asian societies, such as Singapore, Japan, and South Korea, are influenced by Confucianism. Therefore, the people may also have a thinking style similar to *zhongyong* (Nawrot, 2020). In this sense, the implications of the study may not be exclusive to Chinese societies.

In particular, the present study will focus on two research questions: (1) How does PBA affect teacher emotions in the Chinese context? (2) How does ZYT influence the relationship between PBA and teacher emotions in the future?

## Teacher Emotions

According to Schutz et al. (2006), teacher emotions are “socially constructed, personally enacted ways of being that emerge from conscious and/or unconscious judgments regarding perceived successes at attaining goals or maintaining standards or beliefs during transactions as part of social-historical contexts” (p. 344). Similarly, Tsang (2019) suggests that human emotions are the outcomes of continuous negotiations of meanings between social actors who inhabit a set of pre-existing institutional logic regulating their thinking and behaviors. Accordingly, teacher emotions can refer to the feelings of teachers constructed by both their psychological process and the social context in which they exist.

Based on discrete understandings of emotions, education researchers have investigated what kind of emotions teachers usually experience in teaching. For instance, Frenzel (2014) summarizes the common discrete emotions shared by teachers, including enjoyment, pride, anger, anxiety, shame/guilt, boredom, and pity, based on reviewing the literature on teachers’ work and emotions in Western contexts. Nevertheless, findings of Frenzel (2014) may not accurately apply to Chinese contexts because, although Chinese teachers have those emotions, the feeling and expression of emotions are socially and culturally specific (Ekman et al., 1972). Therefore, Chen (2016) focuses her study on teacher emotions in China in an attempt to identify the commonly experienced discrete emotions among Chinese teachers. According to her findings, joy, love, sadness, anger, and fear are the most common teacher emotions in Chinese societies. In particular, Chen (2016) refers to teachers’ joy as to the emotion focused on “positive interactions with students, colleagues, and school leaders” (p.72). Teachers’ love is “teachers’ happiness because of the nature of teaching job such as respect from others, stability, reasonableness of wage, and witness of children’s development” (p.72). Sadness is “teachers feeling unhappy because of ignorance of their efforts, unfair recognition or reward, and students’ unfriendly attitudes” (p.72). Anger is “teachers being annoyed about unfair blame from the public, shifting pressure from school and education bureaucracy, and ignorance of students” (p.72). Fear is “concerned with students’ problems, competition among colleagues, parents’ over-high expectations, and imbalance of life and work” (p.72).

## PBA and Teacher Emotions

Managerial education reforms generally hold schools and teachers accountable for students by different PBA measures, such as using student test performance as an important indicator to evaluate their effectiveness and quality (Saeki et al., 2018). The literature has suggested that PBA tends to make teachers feel negatively about teaching (von der Embse et al., 2017). A possible reason is power. According to the literature, PBA is an institutional force that disempowers teachers to exercise control over their work, leading to an intensifying workload that teachers disvalue (Hargreaves, 2003). Therefore, teachers may find themselves estranged from their professional self, resulting in self-criticism or self-doubt (Kelchtermans, 2016). As a result, teachers may become vulnerable to negative emotions (van Veen et al., 2005).

Accordingly, the present study hypothesizes that PBA is negatively related to Chinese teachers’ emotions of love and joy and positively related to their emotions of anger, sadness, and fear.

## Moderating Role of ZYT

In Chinese societies, people’s thinking style tends to be ZYT because of the influence of a Confucian cultural heritage (Ji and Chan, 2017). ZYT is a culturally dictated thinking style originating from Confucian philosophy rooted in the Doctrine of the Mean. According to this philosophy, a person should be disinclined toward either side and willing to accept no change (Lin et al., 2020). Therefore, it is generally defined as a style of thinking over an issue from multiple perspectives, giving careful consideration to different views, and then making decisions for the sake of both oneself and the general good (Wu and Lin, 2005). According to Wu and Lin (2005), ZYT has three dimensions, including multithinking, holism, and harmoniousness. Multithinking refers to the process of understanding the environment and one’s own needs comprehensively. Holism means the ability to interpret both external and internal factors as a whole. Harmoniousness implies congruous actions by following the guidelines of avoiding extremes. Accordingly, a *zhongyong* thinker tends to consider things carefully, balance all factors comprehensively, avoid extremes, and behave situationally appropriately in an attempt to maintain harmony (Ji et al., 2010).

As teacher emotions are aroused by teachers’ psychological processes, such as thinking style (Frenzel, 2014), they may be affected by ZYT in addition to PBA in the Chinese context. First, multithinking and holism from ZYT would lead teachers to consider both the good and bad cognitively after considering different views in detail (Wu and Lin, 2005). In this regard, teachers with high ZYT are more likely to consider contradictory sides and take multiple perspectives of PBA and developing comprehensive cognitive interpretations (Ji et al., 2000). On the one hand, they feel depressed and anxious about the pressures of PBA. On the other hand, they appreciate the recognition, honor, and material benefits brought about by PBA. Thus, dialectical understanding of PBA help teachers avoid feeling extremely positively or negatively.

Second, harmoniousness in ZYT can function as a self-regulation process (e.g., emotional control) directly, enabling individuals to reduce extreme positive and negative emotions (Pan and Sun, 2018). From the affective perspective, ZYT may influence one’s emotional management because it encourages people to manage their emotions to avoid experiencing or expressing extreme feelings. Accordingly, it is expected that teachers with a high tendency to engage in ZYT may moderate their emotions aroused by PBA.

Therefore, Chinese teachers with high ZYT are more likely to develop complex and paradoxical emotions toward PBA. More specifically, ZYT would weaken the relationship between PBA and teacher emotions from both cognitive and affective perspectives.

## Materials and Methods

### Participants and Procedure

The online survey method was used in this study. The data collection was conducted from March to April 2020. In that period of time, all schools were closed in China because of the COVID-19 pandemic. Therefore, it was difficult for the researchers to go into schools for data collection. To overcome this limitation, they created an online questionnaire with Questionnaire Star, which is a free online platform for questionnaire creation, sent the link to those teachers who they knew in China, and invited them to fill in the questionnaire and forward it to their colleagues. Ultimately, 419 primary and secondary schoolteachers from Hubei, Liaoning, and Beijing participated in the study. After data cleaning, 17 questionnaires were identified as invalid. Therefore, there were 402 (95.94%) valid questionnaires in total. The demographic background of the sample is summarized in Table 1.

**Table 1:** Demographic profile of the participants.

Categories		Frequency	Percentage
Gender	1 = male	117	29.1%
	2 = female	285	70.9%
Age	1 = 30 and below	63	15.7%
	2 = 31–50	281	69.9%
	3 = 50 and above	58	14.4%
Educational attainment	1 = junior college and below	43	10.7%
	2 = bachelor	329	81.8%
	3 = postgraduate	30	7.5%
Professional rank	1 = third grade	23	5.7%
	2 = second grade	52	12.9%
	3 = first grade	196	48.8%
	4 = high grade, senior grade, and special grade	131	32.6%
Teaching experience	1 = 3 years and below	36	9.0%
	2 = 4–20 years	180	44.8%
	3 = 21 years and above	186	46.3%

### Instrument

#### Teacher Emotion

Teacher emotion was measured by the Teacher Emotion Inventory (TEI) developed by Chen (2016). It had 26 items comprising five dimensions, including joy (five items), love (five items), sadness (five items), anger (five items), and fear (six items). Each item was rated from 1 (“never”) to 6 (“almost always”). Examples of items were as follows: “I love to witness my students’ growth”; “I am glad to see my students engage with learning”; “I feel angry when I am treated unfairly (e.g., workload, salary, and appraisal)”; “I feel frustrated when an activity does not work as expected”; and “I am worried to see that my students are pressured by assessments.”

#### Performance-Based Accountability

Performance-based accountability was measured by a three-item scale that has been widely used in previous studies (e.g., Saeki et al., 2018). The items were answered on a five-point Likert scale from 1 (“Not at all”) to 5 (“A lot”). It measured the critical aspects of PBA by assessing the influence of PBA on teachers’ tenure decisions, teacher evaluations, and merit pay. The items were “Student performance on state tests is weighed heavily in tenure decisions,” “Student performance on state tests is weighed heavily in determining my teacher evaluations,” and “Student performance on state tests is weighed heavily in determining my raises/merit/performance pay.”

#### *ZhongYong* Thinking

The ZYT Style Scale was developed by Wu and Lin (2005) to measure ZYT. It consists of 13 items that measured three dimensions of ZYT, including multi-thinking (four items), holism (five items), and harmoniousness (four items). Examples of items were as follows: “When in a discussion, I will consider conflicting opinions at the same time”; “I often try to find acceptable opinions in a situation of disagreement”; and “I usually express conflicting opinions in a tactful way.” All items were rated on a five-point Likert scale from 1 (“strongly disagree”) to 5 (“strongly agree”).

#### Demographic Variables

Previous research has shown that some demographic variables, such as age and gender, may impact emotions (e.g., Brody and Hall, 2008). Moreover, in China’s education system, teachers were professionally ranked by a hierarchical system (Song et al., 2013). According to the system, the teachers could be classified as either third grade (the lowest), second grade, first grade, high grade, senior grade, or special grade (the highest). Professional ranking may affect teachers’ sense of power, which may influence their feelings about teaching (Tsang, 2019). Therefore, these demographic factors are considered the control variables in this study.

### Data Analysis

Confirmatory factor analysis (CFA) was used to test the original PBA, TEI, and ZYT model with maximum likelihood estimation and oblique rotation in Mplus (Costello and Osborne, 2005). During this procedure, some items were deleted in the two models if the regression loading of the item was lower than 0.40 and covariance and variance values were not significant. After that step, a structural equation model (SEM) was employed to examine the relationships between PBA and the five types of teacher emotions and the moderation effect of ZYT on the relationships. It was noted that modification indices were used to modify regression paths. According to current practice, a multicriteria approach for acceptable model fit suggested by Hu and Bentler (1999) was used.

To avoid possible biased estimation caused by the product-indicator approach in the moderation effect test, latent moderated structural equations (LMS) were also conducted. According to Klein and Moosbrugger (2000), “LMS uses the raw data of indicator variables directly for estimation, and does not require the forming of any products of indicator variables” between the predictor, X, and moderator, Z, latent variables to create the latent interaction variable. Thus, LMS was an appropriate approach to test the moderation effect compared with the product-indicator approach (Sardeshmukh and Vandenberg, 2017).

## Results

### Measurement Model: Teacher Emotion Inventory

Based on the criteria above, three items were removed from the TEI in the CFA procedure. The TEI included two positive teacher emotions (joy and love) and three negative teacher emotions (sadness, anger, and fear). The goodness-of-fit index of the 23-item TEI model with five dimensions that proved to be acceptable, *χ^2^* = 757.975; *df* = 218; *χ^2^/df* = 3.48; *CFI* = 0.92; *TLI* = 0.90; *RMSEA* = 0.078; and *SRMR* = 0.074. Five items were used for the joy dimension, which included teachers’ joy on support and recognition from students, parents, colleagues, and school leaders and positive interactions at school. Love included another five items that reflected teachers’ happiness with the teaching profession. Anger consisted of four items that were related to teachers feeling annoyed about unfair treatment and abuse. Sadness (likewise four items) included teachers’ unhappiness due to students’ annoying behavior and attitudes at school. Five items were included in the fear dimension to reflect teachers’ worries and pressures about student achievement, unhealthy competition, and irrational parents.

In Table 2, the CFA results show that all the factor loadings in the TEI were greater than 0.68, indicating strong associations with their relevant constructs. In addition, the item reliability of each factor ranged from 0.85 to 0.93, and the factor reliability was 0.92, in the present study, demonstrating that the items and factors achieved robust reliabilities on each scale, which could be meaningfully used for further analysis.

**Table 2:** Teacher Emotion Inventory (TEI), performance-based accountability (PBA), and *Zhongyong* thinking (ZYT) factors, items, and factor loadings.

Scale and items		Factor loading	Cronbach’s alpha
Teacher Emotion Inventory (TEI)			0.92
Love	I love to witness my students’ growth.	0.71	0.89
	I love to make contributions to my student learning.	0.76	
	I love being a teacher since I can gain a sense of achievement.	0.88	
	I am passionate about the nature of teaching.	0.91	
	I love being a teacher because it is a profession which can obtain respect and recognition from society.	0.76	
Joy	I am glad to see my students engage with learning.	0.94	0.86
	I feel motivated when my students apply what I taught.	0.94	
	I enjoy adopting innovative ideas in my teaching.	0.79	
	I am glad that students enjoy my teaching.	0.63	
	I feel motivated when obtaining support from school leaders.	0.58	
Anger	I feel annoyed when I fail to optimize my students’ learning attitudes.	0.74	0.85
	I feel annoyed when my students do not get along well with me.	0.80	
	I feel angry if my profession has been abused.	0.68	
	I am indignant when the society and/or public blame teachers without any evidence.	0.72	
Sadness	I feel sad when my students behave badly.	0.83	0.93
	I am frustrated if my students do not take ownership for their own learning.	0.92	
	I feel frustrated when the activity design does not work as expected.	0.92	
	I feel frustrated when my professional beliefs are conflicting with the requirements of education reforms.	0.84	
Fear	I am worried about how to improve student achievement.	0.67	0.88
	I feel pressurized from heavy workload (e.g., preparation work).	0.74	
	I am worried about whether I could gain the appropriate opportunities for improvement.	0.77	
	I feel pressurized from the unhealthy competition among colleagues.	0.84	
	I feel pressurized from irrational parents.	0.84	
Performance-based accountability (PBA)			0.82
PBA	Student performance on state tests is weighed heavily on tenure decisions.	0.76	
	Student performance on state tests is weighted heavily in determining my teacher evaluations.	0.94	
	Student performance on state tests is weighted heavily in determining my raises/merit/performance pay.	0.67	
*Zhongyong* thinking (ZYT)			0.93
Multithinking	When in a discussion, I will consider conflicting opinions at the same time.	0.61	0.85
	I tend to think about the same thing from many different perspectives.	0.87	
	I can listen to all the opinions when decisions are made.	0.91	
	When making a decision, I will consider every possible situation.	0.71	
Holism	I often try to find acceptable opinions in a situation of disagreement.	0.63	0.85
	I attempt to find a balance between my opinions and those of others.	0.74	
	I will adjust my original idea after taking into account the views of others.	0.81	
	I anticipate I will acquire common views from discussion.	0.75	
	I attempt to compromise my own opinions from those of others.	0.77	
Harmoniousness	I usually express conflicting opinions in a tactful way.	0.77	0.87
	While deciding on opinions, I attempt to enable the minority to accept opinions of the majority harmoniously.	0.83	
	I usually consider the harmony of the whole when deciding on opinions.	0.74	
	While making my decisions, I usually adjust my method of expression for the sake of the harmony of the whole.	0.83	

### Measurement Model: Performance-Based Accountability

As with the TEI, the three-item model of the PBA was a saturated model that was just identified. The three items included in the PBA model were mainly about the influence and pressure teachers perceived on their tenure decision, performance evaluation, and merit pay. Table 2 also shows that the factor loadings of PBA ranged from 0.67 to 0.94, and the factor reliability was 0.82.

### Measurement Model: *Zhongyong* Thinking

Similarly, the initial 13-item model of ZYT had good model fit compared with the criteria, *χ^2^* = 174.570; *df* = 62; *χ^2^/df* = 2.82; *CFI* = 0.94; *TLI* = 0.93; *RMSEA* = 0.067; and *SRMR* = 0.043. Table 2 also shows that the factor loadings of PBA ranged from 0.67 to 0.94, the factor reliability was 0.82, the item reliability of each factor ranged from 0.85 to 0.87 (0.85, 0.85, and 0.87), and the factor reliability was 0.93 in the present study.

The ZYT comprised three factors, including multi-thinking, holism, and harmoniousness. We did not intend to test the moderation effect of each factor. Rather, all three factors were analyzed as a whole. Therefore, the factorial algorithm method for item parceling was used here to improve the quality of indicators and model fit (Rogers and Neal, 2004).

Table 3 shows the means, SDs, and correlation results of the study variables. The scale correlations among teacher emotions varied from small (−0.04) to large (0.75). Love was highly related to joy (*r* = 0.75^**^, *p* < 0.01), and three negative emotions were highly related to each other, varying from 0.59 to 0.75. Moreover, PBA was significantly positively related to anger (*r* = 0.21^**^, *p* < 0.01), sadness (*r* = 0.23^**^, *p* < 0.01), and fear (*r* = 0.29^**^, *p* < 0.01), which preliminarily supports our proposed hypothesis. Furthermore, the variance inflation factor (VIF) was also assessed to identify any possible multicollinearity among the predictor variables. The results revealed that all VIF values were less than three, indicating that collinearity was not a problem in this study (Dormann et al., 2013).

**Table 3:** Means, SDs, and correlations.

Variables	Means	*SD*	Love	Joy	Anger	Sadness	Fear	PBA	ZYT
Love	5.29	0.81		0.75^**^	0.28^**^	0.15^**^	−0.04	0.05	0.30^**^
Joy	5.48	0.69			0.44^**^	0.28^**^	0.15^**^	0.14^**^	0.36^**^
Anger	4.48	1.21				0.75^**^	0.59^**^	0.21^**^	0.19^**^
Sadness	4.18	1.24					0.66^**^	0.23^**^	0.17^**^
Fear	3.59	1.17						0.29^**^	0.06
PBA	3.58	0.82							0.23^**^
ZYT	4.02	0.51							

** *p* < 0.01.

PBA refers to performance-based accountability, and ZYT refers to zhongyong thinking.

### Structural Model

**Figure 1:**
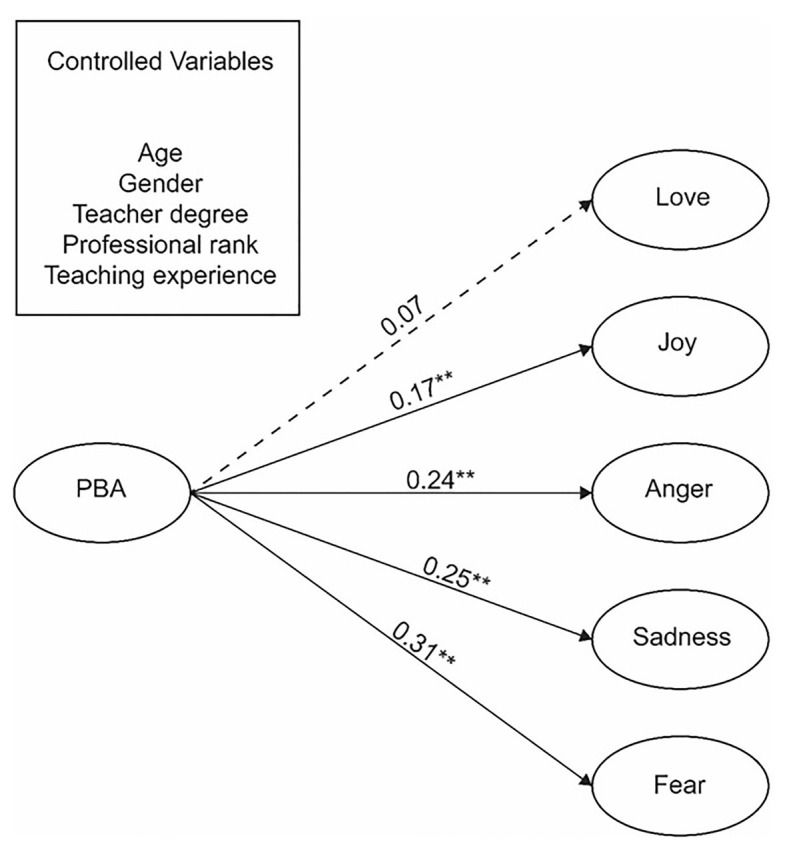
Structural model results showing the path from PBA to teacher emotions. ^**^p < 0.01.

First, a structural model in which all paths from PBA to each TEI factor was tested using standardized estimates. The model indices show acceptable model fit to the data, *χ^2^* = 1241.638; *df* = 492; *χ^2^/df* = 2.52; *CFI* = 0.91; *TLI* = 0.90; *RMSEA* = 0.062; and *SRMR* = 0.059 (Figure 1). Generally, this SEM model portrays that PBA was an influential factor with regard to teacher emotions. More specifically, PBA has a significant explanatory power for negative teacher emotions, and it was significantly positively related to anger, sadness, and fear (*β* = 0.24, *β* = 0.25, *β* = 0.31, *p* < 0.001). The data show that teachers with a higher level of PBA tend to experience more negative emotions, such as anger, sadness, and fear. In contrast, the model results also revealed two interesting findings. First, PBA was not related to love. Second, there was no statistically significant negative relationship between PBA and joy. Instead, PBA was positively and significantly related to joy (*β* = 0.17, *p* < 0.01).

Next, in accordance with the assumption that ZYT would moderate the relationship between PBA and teacher emotions, LMS were used here. However, the result would not report the model fit indices when using the LMS approach in Mplus software. To obtain a sense of how well the model fit, a baseline model that includes the moderator variable was tested in advance using the traditional maximum likelihood estimation procedure. It was after this step that the model with the latent interaction term was estimated, and the Akaike information criterion (AIC) of the two models was compared to judge whether either or both was a well-fitting model (Sardeshmukh and Vandenberg, 2017). Ideally, a smaller AIC means less information loss, suggesting that the optimal model would have the smallest AIC.

**Figure 2:**
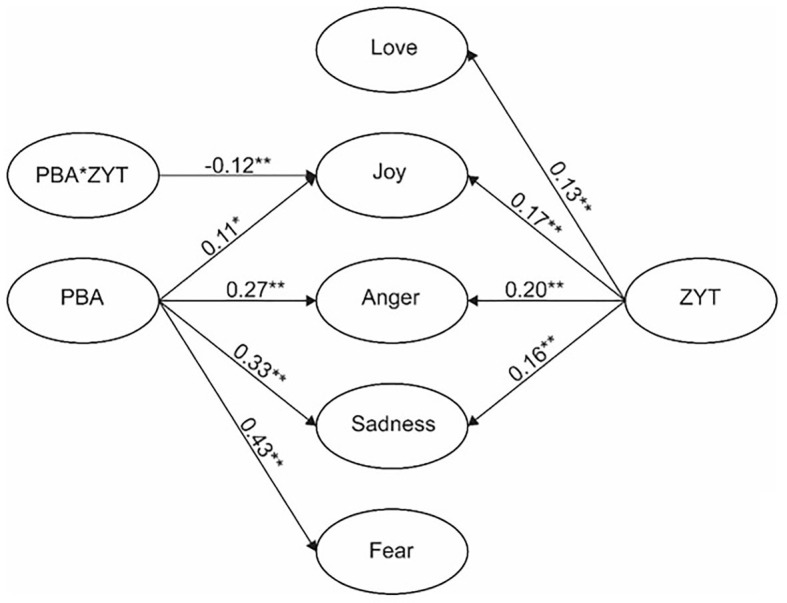
Structural model results showing the moderation effect of ZYT on the relationship between PBA and teacher emotions. PBA refers to performance-based accountability, ZYT refers to *zhongyong* thinking, and PBA * ZYT refers to the interaction of PBA and ZYT. All the reported parameters are standardized. The insignificant path and controlled variables are not shown in the figure. ^*^p < 0.05; ^**^p < 0.01.

**Figure 3:**
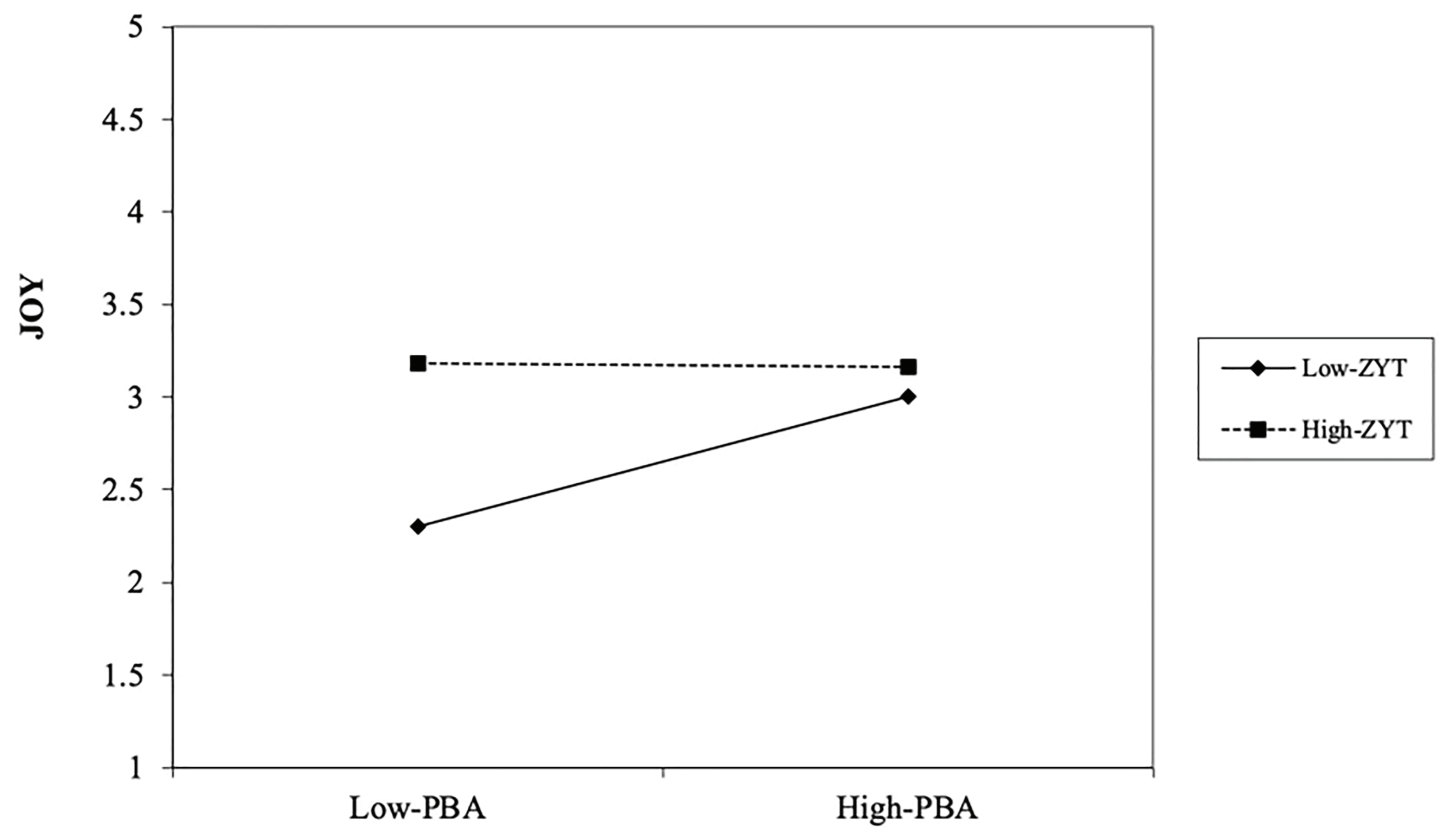
Significant moderating effect of ZYT on the relationship between PBA and Joy. PBA refers to performance-based accountability, and ZYT refers to *zhongyong* thinking.

Before conducting the moderation structural model, the baseline model with the moderation variable (ZYT) was examined. The baseline model had acceptable fit indices, *χ^2^* = 1383.128; *df* = 594; *χ^2^/df* = 2.33; *CFI* = 0.91; *TLI* = 0.90; *RMSEA* = 0.057; and *SRMR* = 0.059. The AIC of the baseline model was 25946.198. Then, the moderation model, which included the latent interaction term, was estimated (Figure 2), and the AIC for the latter model was 25946.604. According to Burnham and Anderson (2002), the difference in the AIC (*Δ*_i_ = AIC_i_ – AIC_min_) can be very important and useful in determining the best model. *Δ*_i_ = 4 – 7 can indicate that the model with the smaller AIC has considerably better fit, and *Δ*_i_ > 10 can rule out the worse-fitting model. In the present study, *Δ*_i_ = 0.406, suggesting little information loss and indicating that the model with the latent interaction term is also acceptable like the baseline model. As shown in Figure 2, PBA was a predictor of three negative emotions and the joy scale, whereas ZYT also positively predicted love, joy, anger, and sadness. More importantly, the latent interaction term (PBA * ZYT) negatively predicted joy (*β* = −0.12, *p* < 0.01), which indicated that ZYT moderated the original positive relationship between PBA and joy (see Figure 3). This result indicated that teachers with high levels of ZYT were likely to experience less joy than teachers with low levels of ZYT in the same PBA context.

## Discussion

Similar to previous studies on teacher emotions in Western societies (e.g., Hargreaves, 2003; Kelchtermans, 2016), the research findings suggest that PBA may also cause teachers to experience emotions of anger, sadness, and/or fear in China. According to the literature, PBA may disempower teachers to control their work and subject them to external monitoring regarding their performance on the heavy workload that they disvalue (Tsang, 2019). Therefore, teachers may feel anger, sadness, or fear toward the teaching environment because they are accountable for many duties that may be meaningless to them (Hargreaves, 2003).

First, we will discuss the direct effects of PBA on teacher emotions separately. Different from the literature, the findings show that PBA may lead to teacher joy and negative emotions in China. To explain the unexpected findings, it is necessary to consider the sociocultural context of China’s education system. Owing to the long tradition of examination-oriented systems in China (Miyazaki, 1974), there is a fundamental and strong cultural recognition of test performance for Chinese teachers. Therefore, preparing students for good performance in examinations may become a commonly used pedagogy for many teachers in Chinese societies (Pong and Chow, 2002). Moreover, the tight relationship between student performance and teachers’ own benefits, professional recognition, and social status encourages teachers to try their best to reach excellent performance goals in teaching. For example, the Chinese government initiated a teacher honor system that ranks teachers in terms of their teaching quality, which is defined by their students’ attainment in examinations (Lo et al., 2015). The highly ranked teachers are further awarded an honorary title at the school, municipal, provincial, and/or national level, and thus, are perceived as the most professional and best teachers in society, leading to high self-esteem, dignity, and a sense of achievement (Song et al., 2013). Moreover, the performance-based teacher salary reform implemented in 2009 emphasized the principle of “good performance, good pay” in 2009 (Wang et al., 2014). In this context, although Chinese teachers may be accountable to students’ examination performance and experience pressure to improve their students’ test performance, they may still welcome PBA measures (Liu et al., 2016). PBA provide opportunities for them to earn social recognition, status, and financial rewards if they can satisfy the performance indicators. Therefore, it is possible for PBA to help teachers feel joy in China.

Second, we will discuss the moderation effect of ZYT on the relationship between PBA and joy in order to deepen our understanding of the relationship between the three variables. As expected, the study indicates that ZYT may moderate the relationship between PBA and teacher emotions, especially joy. To some extent, the findings imply that ZYT as a psychological process may play a role in teacher emotional arousal. Specially, there is a positive relationship between PBA and joy for teachers with a lower tendency toward ZYT. However, the positive relationship does not exist for teachers with a higher tendency toward ZYT. There are two possible explanations to the finding. First, teachers with a higher level of ZYT tends to realize the side effects of PBA on students’ overall development (Yin, 2008) or teachers’ professionalism (Wang et al., 2014), because of the multithinking and holism. Second, it is also possible that they are inclined to emotionally adjust themselves and avoid feeling and expression of extreme emotions that may disrupt social harmony (Ji et al., 2010). Accordingly, these may be the reason why the present study indicates ZYT as a moderator suppressing the positive relationship between PBA and joy among Chinese teachers.

A limitation of the study is the sampling method. This study only collected cross-sectional data through a snowball sampling method owing to the COVID-19 pandemic, leading to a weak causal analysis and a potential sampling bias. Therefore, future studies are recommended to use longitudinal study design and replicate the present study with probability samples. Moreover, the demographic variables are only used as control variables in this study, and we propose to use them as independent variables to test their effects on the relationships among PBA, ZYT, and teacher emotions. Lastly, there may be some cultural differences between China and other Chinese societies, such as Hong Kong, Taiwan, and Singapore. Therefore, the research findings may not represent other sections of Chinese society. It is necessary to study the effects of ZYT on teacher emotions there as well.

## Conclusion

The main purpose of the present study was to identify Chinese teachers’ emotions toward PBA in a special examination-oriented cultural context first. Moreover, we also explored the moderation effect of ZYT on the relationship between PBA and teacher emotions. To conclude, the present study showed that PBA is an significant factor with respect to teachers’ joy, sadness/frustration, anger, and fear, as related to their job of teaching, but not love of their profession. The second major finding was that the ZYT may moderate the relationship between PBA and joy. According to the study, there are some implications to teacher education. As influenced by ZYT, teachers with a higher level of ZYT are more likely to manage their emotions and conform to PBA even though they are dissatisfied with it, leading to more rational knowledge and comprehensive understandings. Therefore, it is recommended that teacher education prepare teachers to become reflexive agents who can holistically interpret an issue from different perspectives, insist on their pedagogical standpoint, and innovatively respond to or copy any challenges. As a reflexive agent, therefore, teachers with ZYT may become more capable of maintaining social harmony, even though they express resistance or negative feelings to unjust PBA measures. Thus, teacher education should go beyond training subject content knowledge, curriculum design, and teaching skills but encourage teachers to engage in forms of self-study with emphasis on improving their professionalism through reflexive methods (Moore, 2010). For example, recent studies suggest that writing practicum portfolios may help preservice teachers reflect on the relationship between pedagogical theories and their teaching experiences in the practicum to facilitate them in developing a more holistic conception of teachers’ work (Körkkö et al., 2016). Moreover, To (2020) suggests that photovoice, a method that requires people to reflect by taking photos regarding particular lived experiences, can encourage teachers to make critical reflections on their work life and in turn facilitate them to have new insights about the meanings of teaching and education. Therefore, teacher educators can incorporate these or similar methods in teacher education courses and programs.

## Data Availability Statement

The original contributions presented in the study are included in the article/supplementary material, further inquiries can be directed to the corresponding author.

## Ethics Statement

The studies involving human participants were reviewed and approved by Research Ethics Committee, College of Educational Administration, Beijing Normal University. The patients/participants provided their written informed consent to participate in this study.

## Author Contributions

YZ contributed to the study design, data collection, and data analysis. The Materials and Methods and the Results sections were written by YZ, and the Introduction and the Discussion sections were written by KT. The rest of sections were drafted by YZ and revised by KT. Both the authors contributed to the article and approved the submitted version.

### Conflict of Interest

The authors declare that the research was conducted in the absence of any commercial or financial relationships that could be construed as a potential conflict of interest.
